# Both anti-inflammatory and antiviral properties of novel drug candidate ABX464 are mediated by modulation of RNA splicing

**DOI:** 10.1038/s41598-018-37813-y

**Published:** 2019-01-28

**Authors:** Audrey Vautrin, Laurent Manchon, Aude Garcel, Noëlie Campos, Laure Lapasset, Abdelhamid Mahdi Laaref, Roman Bruno, Marie Gislard, Emeric Dubois, Didier Scherrer, J Hartmut Ehrlich, Jamal Tazi

**Affiliations:** 1 IGMM, CNRS, Univ. Montpellier, Montpellier, France; 2 ABIVAX, 1919 route de Mende, 34293 Montpellier Cedex 5, Montpellier, France; 3 ACOBIOM, 1682 Rue de la Valsière, 34184 Montpellier Cedex 4, Montpellier, France; 4 MGX, Univ Montpellier, CNRS, INSERM, Montpellier, France

## Abstract

ABX464 is a first-in-class, clinical-stage, small molecule for oral administration that has shown strong anti-inflammatory effects in the DSS-model for inflammatory bowel disease (IBD) and also prevents replication of the HIV virus. ABX464 which binds to cap binding complex (CBC) has demonstrated safety and efficacy in a phase 2a proof-of-concept clinical trial in patients with Ulcerative colitis. Previously, with limited technologies, it was not possible to quantify the effect of ABX464 on viral and cellular RNA biogenesis. Here, using RNA CaptureSeq and deep sequencing, we report that ABX464 enhances the splicing of HIV RNA in infected PBMCs from six healthy individuals and also the expression and splicing of a single long noncoding RNA to generate the anti-inflammatory miR-124 both *ex vivo* and in HIV patients. While ABX464 has no effect on pre-mRNA splicing of cellular genes, depletion of CBC complex by RNAi leads to accumulation of intron retention transcripts. These results imply that ABX464 did not inhibit the function of CBC in splicing but rather strengthens it under pathological condition like inflammation and HIV infection. The specific dual ability of ABX464 to generate both anti-inflammatory miR-124 and spliced viral RNA may have applicability for the treatment of both inflammatory diseases and HIV infection.

## Introduction

ABX464 is a novel drug candidate for treating patients infected with human immunodeficiency virus (HIV) and patients with ulcerative colitis (ABIVAX, data in file). Despite the successful control of viremia, many HIV-infected individuals treated with ART exhibit residual inflammation associated with non-AIDS-related morbidity and mortality. Several reports have shown that measures of inflammation and immune activation are the best independent predictors of disease progression in HIV-infected individuals. Thus, the anti-inflammatory activity of ABX464 is potentially relevant for the intended use in treating HIV patients, in whom the inflammation around viral reservoirs was shown to substantially contribute to adverse cardiovascular and tumorigenic effects despite long-term ART-treatment. In addition, ABX464 protects mice from the lethal effects of DSS (Dextran Sulphate Sodium), which is a key animal model for inflammatory bowel disease^1^. Patients with UC may benefit from ABX464 which has demonstrated safety in phase 2 clinical trial (ABIVAX, data in file) and has a mode of action different from classical medications including corticosteroids, immunomodulators and biologic treatments.

ABX464 is a small molecule that binds to the cap binding complex (CBC)^2^, a complex at the 5′-end of the pre-mRNA transcript that promotes the initial interaction with transcription and processing machinery^3–5^. The CBC recruits several factors to m7G-modified transcripts to mediate processing events and is required for efficient cellular and viral pre-mRNA splicing^3^. The interaction of CBC with the U1 snRNP at the 5′ splice site of the first intron in the transcript^4,6^ and direct interaction of CBC with proteins in U4/U5/U6 particles enhances the formation of spliced mRNAs^5,7^. Although CBC is not essential for viability in either yeast or humans^8,9^, its deletion results in a reduction in the recruitment of several splicing factors to the nascent transcript, resulting in inhibition of cotranscriptional spliceosome assembly^5^. The CBC complex has also been shown to affect microRNA biogenesis^10–12^. miRNAs are transcribed by RNA pol II as primary (pri)-miRNAs, which carry the m7G cap^13^. During nuclear and cytoplasmic processing events, the pri-miRNA loses the m7G cap, and the mature, 21–23-nucleotide-long miRNA is incorporated into RISC (RNA-induced silencing complex) to guide RNA silencing^12^. Since a large fraction of miRNA genes are located in introns^14,15^, the CBC complex may be involved in the interplay between the processing of intronic pre-miRNAs and pre-mRNAs^16,17^.

ABX464 inhibits viral replication by affecting the biogenesis of viral RNA^2^ but its effect on cellular and viral RNA biogenesis has not been analyzed in detail. ABX464 will only act on viral replication once proviral DNA was integrated to cellular DNA. This is important as the viral genome, once integrated in infected cells, requires both activation and inhibition of precursor mRNA splicing^18,19^. Successful infection and production of new infectious HIV particles requires the balanced expression of seven viral proteins (Rev, Tat, Nef, Vif, Vpr, Vpu and Env) that are produced by splicing of the HIV-1 primary 9 kilobases (kb) transcript; among these, the Tat and Rev factors are essential for viral gene expression at the transcriptional and posttranscriptional levels in infected cells^18,19^. The HIV-1 primary transcript serves not only as genomic RNA for progeny virus but also as the mRNA that encodes the viral Gag and Gag-Pol proteins^18,19^. While most cellular unspliced RNAs are retained in the nucleus, where they are degraded^20^, nuclear export of the unspliced viral RNAs is facilitated by the Rev protein through binding to the Rev responsive element (RRE)^21–24^ and interaction with CRM1-dependent export machinery^23,25^. Therefore, inefficient alternative splicing is required to maintain a balance between HIV gene expression and viral production^18,26^. This balance is thought to be mediated by the HIV long terminal repeat (LTR) and the presence of suboptimal viral 5′ and 3′ splice sites (5′ and 3′ ss), which are positively regulated by regulatory sequences and their recognition by cognate trans-acting cellular factors^26–32^. By binding the CBC complex, ABX464 has been shown to interfere with Rev-mediated export of unspliced RNA^2^. However, the underlying mechanisms behind modulation of viral and cellular splicing and/or miRNA biogenesis by the binding of ABX464 to CBC are presently unknown.

In this study, we elucidate the mechanism of action of ABX464 as small molecule to treat patients with HIV infection and IBD. Using RNA capture and deep sequencing we demonstrate that both effects are mediated through the same mechanism i.e. enhanced pre-mRNA splicing. ABX464 not only enhances pre-mRNA splicing of HIV viral RNA to block HIV replication but also triggers the splicing of a long non-coding RNA which houses one of the loci for the anti-inflammatory miR-124 and thereby increases the expression of the anti-inflammatory microRNA, miR-124. Our findings may open up new treatment options for patients with HIV and inflammatory diseases.

## Results

### ABX464 generates spliced HIV RNA variants

To profile viral transcriptional events modulated by ABX464 and thereby assess the full depth of the HIV transcriptome, we employed a recently described targeted RNA capture and sequencing strategy (RNA CaptureSeq). This strategy involves the construction of tiling arrays across the HIV genome, against which cDNAs are hybridized, eluted and sequenced. RNA CaptureSeq is similar to previous in-solution capture methods^33^ and exome sequencing approaches^34^, but when combined with deep-sequencing technology, provides saturating coverage and permits the robust assembly of rare and unannotated HIV transcripts^35^.

**Figure 1:**
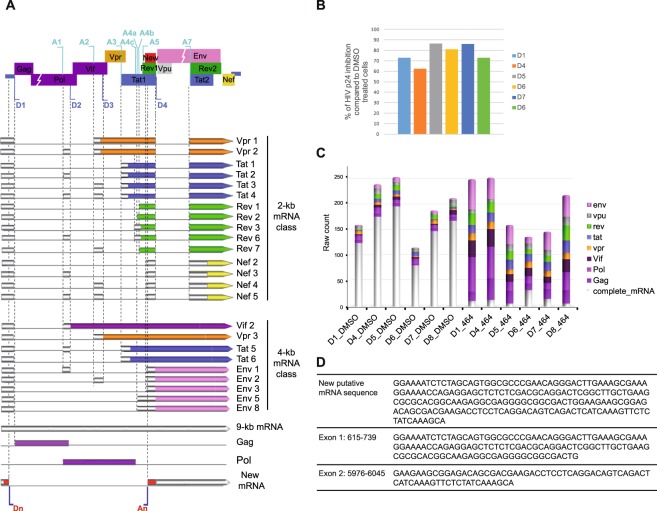
Analysis of HIV splicing following ABX464 treatment. (**A**) Organization of the HIV-1 genome and different mRNA splicing products. The 5′ ss (D1–D4) and 3′ ss (A1–A7) are indicated. ORFs of the coding exons of each mRNA product are indicated with a different color code indicating the corresponding encoded proteins of the HIV genome. The non-coding exons are boxed in gray. (**B**) Inhibition of HIV-1 replication measured by p24 production in PBMCs from six donors. (**C**) Quantification of HIV splicing events using RNA CaptureSeq in PBMCs from six different donors that were infected and untreated (DMSO) or treated with ABX464 for 6 days (464). Counts of spliced and unspliced contigs are shown. The different splicing products are colored as in A. (**D**) Sequence of the new viral RNA generated by splicing.

PBMCs from 6 HIV-negative donors were infected with the YU-2 strain, followed by treatment with ABX464. Our protocol resulted in mild infection that did not lead to cell death at 8 days postinfection (dpi), and viral replication was inhibited by more than 70% with 5 µM of ABX464 (Fig. 1B). After cDNA capture from infected cells that were untreated or treated with ABX464, libraries were prepared and sequenced using an Illumina sequencing facility. To highlight potential new splicing events induced by ABX464, we used a custom bioinformatics pipeline with the main step involving the assembly of putative transcripts from targeted RNA sequencing reads to construct contigs (Illumina paired-end 2 × 75 bp) (Fig. S1A,B). The putative transcripts (contigs) were then mapped to the HIV genome. The HIV profiling datasets used in our analysis yielded high sequence coverage (2 to 30 million reads for the 9 kb genome), allowing deep quantification of HIV splice variants. We were able to reliably quantify each HIV alternative splicing over a wide range of transcript abundances and show that while ABX464 treatment resulted in enhanced splicing, ABX464 did not favor generation of any splice variant over the others (Fig. 1C, Table S1).

To test whether enhanced splicing by ABX464 generates new viral RNA variants, the splicing events were filtered and analyzed. After mapping the contigs to the genome of the YU-2 strain, the latter was used as an anchor for additional clustering and assembly of new HIV transcripts. The partial gene structures generated by the spliced alignment were merged whenever they shared consecutive splicing sites spanning an intronic viral sequence. The superstructures so formed correspond to all possible HIV gene structures for which each complete exon is supported by at least one of the alignments (Supplementary Materials, Materials and Methods). At the HIV transcript level, this led to the formation of superassemblies, where each superassembly is a merge of initial contigs matching a predicted superstructure. Notably, the linking of contigs to the HIV-1 genomic sequence and the requirement that all splicing sites in merged contigs coincide, precluded the formation of spurious superassemblies. Figure 1C and Table S1 present the distribution of the number of contigs that were merged to form one superassembly for each donor. While, in all samples treated by ABX464, most of the assembled contigs (90%) corresponded to spliced RNAs (Fig. 1C, Table S2 and Fig. S2) or small RNAs containing gag-pol sequences that can be released from introns, in untreated samples, spliced RNAs represented a minor fraction (less than 24%), and the majority of contigs corresponded to full-length unspliced viral RNA (more than 74%) (Fig. 1C, Table S2 and Fig. S2). This result demonstrated that ABX464 preferentially generated spliced HIV RNA variants in infected PBMCs, which would compromise the subsequent synthesis of full-length HIV-1 pre-mRNA and assembly of infectious particles, thereby leading to inhibition of viral replication.

Interestingly, one of the spliced variants generated by ABX464 treatment (Fig. 1A,D) was present in cells infected by both the YU-2 and Ada-M strains, and no polymorphism in its sequence was detected in the HIV-1 B and C subtypes that were strongly inhibited by ABX464. Furthermore, this new splice variant was detected using long read sequencing, which points to the additional accuracy that this method brings in delineating complex and rare spliced isoforms and estimating their relative abundances^36^. This new RNA variant can generate immunogenic peptides or being toxic to cells harboring proviral DNA. This is consistent with the diminished viral loads and reduced viral DNA levels (ABIVAX, data in file) observed in HIV patients treated with ABX464^37^. Thus, ABX464 not only enhanced the splicing of HIV RNA but also generated a new HIV splice variant.

### ABX464 does not affect cellular splicing

**Figure 2:**
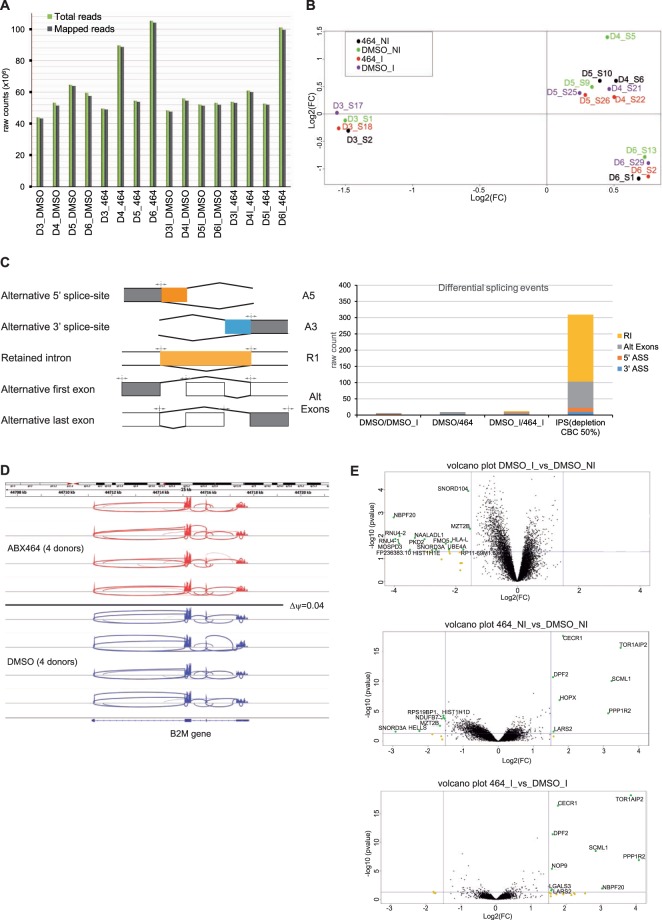
ABX464 has no global effect on cellular splicing. (**A**) The effect of ABX464 on infected and uninfected CD4+ T cells was tested by a high-throughput RNAseq approach. Sixteen libraries were constructed using 4 conditions: uninfected (DMSO_NI), uninfected treated with ABX464 (ABX464_NI), infected (DMSO_I) and infected treated with ABX464 (464_I), corresponding to 4 donors. Approximately 38 million reads (more than 50% of the total raw reads) were aligned to exons of the human genome sequence in each of the samples. (**B**) Multidimensional scaling analysis (MDS) was used to interpret major trends in the data. (**C**) Alternative splicing (AS) events of cellular genes were classified into five major groups (left panel): Alternative 5′ splice site (A5SS, orange), Alternative 3′ splice site (A3SS, blue), Skipped exon (SE, gray), Mutually exclusive exons (MXE, gray) and Retained exon (RI, yellow). AS event counts comparing infected vs uninfected samples (DMSO_I vs DMSO_NI), uninfected vs uninfected treated by ABX464 (DMSO_NI vs 464_NI), infected vs infected treated by ABX464 (DMSO_I vs 464_I) and after 50% depletion CBC in IPS (IPS depletion of CBC by 50%) (right panel). (**D**) By comparing exon coverage reads of a common highly expressed gene (*B2M*) between the ABX464 and DMSO conditions in the 4 donors, we confirmed that ABX464 did not increase splicing events in *B2M*. (**E**) Volcano plot of DMSO_I vs DMSO_NI (upper panel), DMSO_NI vs 464_NI (middle panel), DMSO_I vs 464_I (lower panel). The gene expression variation generated by ABX464 treatment was very low in infected (6 downregulated genes) and uninfected (6 downregulated genes) samples.

To ensure that ABX464 acted specifically on HIV splicing and did not significantly or globally affect the splicing events of human genes, we used a high-throughput RNAseq approach. Many genome-wide expression studies of HIV infection are based on analyses of total peripheral blood mononuclear cells (PBMCs)^38,39^, which consist of over a dozen cell subsets, including T cells, B cells, NK cells and monocytes. To avoid the dilution of the specific gene expression signals of particular cell subsets by those from the other cells and thus a reduction of the specificity of this approach, we used purified CD4+ T cells from the PBMCs of 4 donors. The CD4+ T cells were uninfected or infected with the YU-2 strain and were untreated or treated for 6 days with ABX464, followed by high-throughput RNAseq. Each raw dataset of the samples contained between 44 and 105 million single-end reads (50 bp), with an average of approximately 60 million raw reads per sample (Fig. 2A). More than 97% of the bases had a quality score of ≥Q20. Approximately 98% of the total raw reads were mapped to the human genome sequence (GRCh38), giving an average of 60 million human reads per sample for further analyses. The reads that were correctly mapped (approximately 98% of total input reads) to the gene and transcript locations (GTF annotation file) (Fig. 2A) were subsequently analyzed using in-house package suites for transcript abundance normalization and evaluation. Multidimensional scaling analysis (MDS) is an unsupervised global analysis approach and is useful for reducing the dimensionality of gene expression data (Fig. 2B). MDS minimizes dimensions, preserving the distances among data points, and allows for projecting multidimensional gene data onto just two or three new dimensions that explain most of its variance. As a result, it is possible to visually interpret major trends in the data, for example, in terms of the similarities among various data points. The MDS of our gene expression data showed, without any outliers, that the different donors separated well and distributed into the DMSO (untreated) and ABX464 treatments that were infected or uninfected. The displayed variance was donor-dependent (clustered by donor) but treatment-independent (no data structure related to the different treatments), which suggests that the ABX464 molecule did not induce a major difference in CD4+ T cell gene expression (Fig. 2B).

For a gross estimation of the alternate splicing events modulated by ABX464 in infected and uninfected CD4+ T cells, we compared junction read counts that originated from exon-exon boundaries across different samples (Fig. 2C). While the alignment of reads to exon junctions was purely coincidental, we did not observe gross differences in the total number of reads corresponding to exon-exon boundaries between treated and untreated CD4+ T cells, whether infected or uninfected, across any of the samples (Fig. 2C). For a more statistically qualified assessment of alternate splicing in these CD4+ T cells, we followed a method for differential splicing analysis across multiple conditions named SUPPA (super-fast pipeline for alternative splicing analysis)^40^. The alternate splicing events were classified into five major groups: Alternative 5′ splice site (A5SS), Alternative 3′ splice site (A3SS), Alternative first and last exons (Alt exons) and Retained exon (RI) (Fig. 2C). A “percent splicing index (psi)” score (ψ-score) was calculated for each of the transcript variants for each sample^41^. Using a stringent cutoff, the difference of ψ-score from untreated sample was fixed at 0.4 and *p*-value was fixed at 0.05 for differential splicing events induced upon treatment with ABX464 to be considered significant. The number of significant events for each of the 5 possible AS events is presented in Fig. 2C and Table 1. No switch-like events were detected where the difference in psi-score was exactly 1 or −1. We next calculated the ψ-scores of transcripts in uninfected versus infected CD4+ T cells (Fig. 2C). No switch-like events were detected in infected or uninfected T CD4+ samples when compared to ABX464-treated samples (Fig. 2C). The exact numbers of common and differential splicing events between infected and uninfected CD4+ T cells treated with ABX464 were very low (fewer than 10 events, Table 1), while depletion of NCBP1 (Nuclear Cap Binding protein subunit 1), a component of the CBC, in stem cells by only 50% gave rise to large variations, with 81 Alt exon, 12 A 5′ SS, 10 A 3′ SS and 206 IR events (Fig. 2C, Data File S1). The high level of IR indicated that CBC complex is a major component preventing accumulation of unspliced RNA as most of unspliced transcripts will be degraded by nonsense mediated decay (NMD). Comparison of exon coverage reads of a common highly expressed gene, *B2M*, between the ABX464 and DMSO conditions in the 4 donors revealed that ABX464 did not increase splicing events in *B2M* (Fig. 2D). In summary, ABX464 treatment did not induce alternate splicing of transcripts, and therefore, ABX464 had no potential to dramatically alter gene expression in activated CD4+ T cells. Consistent with this result, FACS analysis of purified activated CD4+ T cells or PBMCs from 7 donors after 6 days of ABX464 treatment did not reveal any changes in the CCR6/CXCR3 or CD45/CCR7 (Th17/Th1 and effector memory cells, respectively) subpopulation (Fig. S3).

**Table 1:** Splicing events in CD4 T cells: infected vs uninfected (DMSO_I vs DMSO), uninfected treated with ABX464 vs uninfected and untreated (464 vs DMSO) and infected treated with ABX464 vs infected and untreated (464_I vs DMSO_I).

ABX464_NI_VS_DMSO_NI			
Gene symbol	Ensembl ID	description	Event type
LINC01119	ENSG00000239332	long intergenic non-protein coding RNA 1119	A3
STMND1	ENSG00000230873	stathmin domain containing 1	Alt exons
ZNF177	ENSG00000188629	zinc finger protein 177	A3
FGF22	ENSG00000070388	fibroblast growth factor 22	A3
AL139035.1	ENSG00000280710	Clone-based	Alt exons
MRPL52	ENSG00000172590	mitochondrial ribosomal protein L52	A5; Alt exons
HCG20	ENSG00000228022	HLA complex group 20 (non-protein coding)	Alt exons
RHBDD2	ENSG00000005486	rhomboid domain containing 2	Alt exons
**ABX464_I_VS_DMSO_I**			
AL157955.2	ENSG00000258826	Clone-based	A3
TMEM147	ENSG00000105677	transmembrane protein 147	RI
COX6C	ENSG00000164919	cytochrome c oxidase subunit 6C	Alt exons
ATIC	ENSG00000138363	5-aminoimidazole-4-carboxamide ribonucleotide formyltransferase/IMP cyclohydrolase	A5
STMND1	ENSG00000230873	stathmin domain containing 1	Alt exons
GEM	ENSG00000164949	GTP binding protein overexpressed in skeletal muscle	Alt Exons
CDRT15	ENSG00000223510	CMT1A duplicated region transcript 15	A5
WDR72	ENSG00000166415	WD repeat domain 72	A5
SMIM11A	ENSG00000205670	small integral membrane protein 11A	AL
EGLN2	ENSG00000269858	egl-9 family hypoxia inducible factor 2	Alt exons
GNRHR2	ENSG00000211451	gonadotropin releasing hormone receptor 2 (pseudogene)	RI
ZNF584	ENSG00000171574	zinc finger protein 584	A3
LINC01869	ENSG00000180279	long intergenic non-protein coding RNA 1869	A3
**DMSO_I_VS_DMSO_NI**			
CHTF8	ENSG00000168802	chromosome transmission fidelity factor 8	Alt exons
KCTD2	ENSG00000180901	potassium channel tetramerization domain containing 2	A5
ETS1	ENSG00000134954	ETS proto-oncogene 1; transcription factor	A5
RASAL2-AS1	ENSG00000224687	RASAL2 antisense RNA 1	A5
SLC16A9	ENSG00000165449	solute carrier family 16 member 9	Alt exons
RPP40	ENSG00000124787	ribonuclease P/MRP subunit p40	Alt exons

### ABX464 does not alter cellular gene expression

To determine whether ABX464 induced a quantitative change in the transcriptome of infected cells, we measured transcript levels (see Fig. S1 for pipeline) from the RNAseq data. More than 60 000 different transcripts were detected among all samples. After counts per million normalization (CPM), transcripts of each sample were filtered out with a coverage cutoff value of CPM >5; that is, a gene was considered to be expressed in a sample if it had at least five counts for each million mapped reads in that sample. Since this experiment used four donors, four replicates were available for each condition (infected/uninfected cells with/without ABX464 treatment), and a gene was considered to be expressed if it was expressed to at least five counts for each million mapped reads in all replicates. Following these selection criteria, we ended up with a list of transcripts common to all samples, including 11 700 different transcripts with an average of around 3 000 raw counts per transcript. Using the CPM values of the qualified genes, a global expression plot was produced for each donor. Under the conditions of mild infection used in our study, we did not detect strong changes in gene expression, with only 15 genes being downregulated in response to infection in untreated samples (Fig. 2E top panel, Table 2). ABX464 treatment resulted in the upregulation of 9 genes in the infected samples, and 6 downregulated and 7 upregulated genes in the uninfected samples (Fig. 2E middle and lower panels, respectively; Table 2), demonstrating a mild effect of ABX464 treatment on gene expression (Fig. 2E). The six genes that were upregulated following ABX464 treatment were shared between infected and uninfected samples, suggesting that this upregulation was mediated by ABX464 treatment and was independent of infection. Three of the six genes, *TOR1AIP2*, *SCML1* and *PPP1R2*, had fold changes above 3 with *p*-values of 10^−5^. Interestingly, TOR1AIP2, a protein thought to regulate protein folding as well as intracellular trafficking, was recently shown to constrain the late steps of HIV replication^42^ and may, therefore, be an effector of ABX464-induced changes.

**Table 2:** Genes modulated in T CD4 cells: infected vs uninfected (DMSO_I vs DMSO), uninfected treated with ABX464 vs uninfected and untreated (464 vs DMSO) and infected treated with ABX464 vs infected and untreated (464_I vs DMSO_I).

Gene Symbol	Ensembl ID	chr	description	log2(FC)	FC	P-Value
SNORD104	ENSG00000199753	17	small nucleolar RNA, C/D box 104	−1,593239134	0,3314265	0,000113748
MZT2B	ENSG00000152082	2	mitotic spindle organizing protein 2B	−1,545615646	0,342549493	0,005014214
HLA-L	ENSG00000243753	6	major histocompatibility complex, class I, L (pseudogene)	−2,155554923	0,224446745	0,018996735
FMO5	ENSG00000131781	1	flavin containing monooxygenase 5	−2,32723033	0,199266303	0,022279359
RP11-69M1.6	ENSG00000279865	12	pseudogene	−1,75254756	0,296777256	0,040623948
UBE4A	ENSG00000110344	11	ubiquitination factor E4A	−2,226441782	0,2136851	0,039977521
SNORD3A	ENSG00000263934	17	small nucleolar RNA, C/D box 3A	−2,648926643	0,159438656	0,041651979
HIST1H1E	ENSG00000168298	6	histone cluster 1 H1 family member	−2,769812645	0,146623409	0,043246739
PKD2	ENSG00000118762	4	polycystin 2, transient receptor potential cation channel	−3,020380563	0,123246573	0,014543367
NAALADL1	ENSG00000168060	11	N-acetylated alpha-linked acidic dipeptidase like 1	−3,333148179	0,099225299	0,012581
RNU4-1	ENSG00000200795	12	RNA, U4 small nuclear 1	−3,972391545	0,063707563	0,014002979
RNU4-2	ENSG00000202538	12	RNA, U4 small nuclear 2	−3,830896068	0,070272495	0,010356151
MOSPD3	ENSG00000106330	7	motile sperm domain containing 3	−3,845633809	0,069558286	0,022670456
FP236383.10	ENSG00000277105	8	miRNA	−3,494083339	0,088751583	0,040882842
NBPF20	ENSG00000162825	1	Neuroblastoma Breakpoint Family, Member 20	−4,017571383	0,061743395	0,001624932
**ABX464_NI_vs DMSO_NI**						
SNORD3A	ENSG00000263934	17	small nucleolar RNA, C/D box 3A	−2,923985868	0,13176	0,026481173
HELLS	ENSG00000119969	10	helicase, lymphoid specific	−2,246421514	0,21075	0,018558217
MZT2B	ENSG00000152082	2	mitotic spindle organizing protein 2B	−1,658291634	0,31681	0,002657817
HIST1H1D	ENSG00000124575	6	histone cluster 1 H1 family member d	−1,559586171	0,33925	5,18E-05
RPS19BP1	ENSG00000187051	22	ribosomal protein S19 binding protein 1	−1,566230636	0,33769	0,000115799
NDUFB7	ENSG00000099795	19	NADH:ubiquinone oxidoreductase subunit B7	−1,520475764	0,34857	0,000168054
CECR1	ENSG00000093072	22	Adenosine Deaminase 2	1,842665068	3,58672	2,65E-18
DPF2	ENSG00000133884	11	double PHD fingers 2	1,560531204	2,94962	2,45E-11
HOPX	ENSG00000171476	4	HOP Homeobox	1,744005457	3,34964	1,47E-07
LARS2	ENSG00000011376	3	leucyl-tRNA synthetase 2, mitochondrial	1,580307045	2,99033	0,026767164
TOR1AIP2	ENSG00000169905	1	torsin 1A interacting protein 2	3,485915121	11,20379	2,59E-16
SCML1	ENSG00000047634	X	Scm polycomb group protein like 1	3,229297195	9,37811	9,67E-11
PPP1R2	ENSG00000184203	3	protein phosphatase 1 regulatory inhibitor subunit 2	3,136231549	8,79224	2,52E-05
**ABX464_I_vs_DMSO_I**						
CECR1	ENSG00000093072	22	Adenosine Deaminase 2	1,770654479	3,41208711	4,33E-17
DPF2	ENSG00000133884	11	double PHD fingers 2	1,61885639	3,071314797	4,52E-12
NOP9	ENSG00000196943	14	NOP9 nucleolar protein	1,594464835	3,019824765	4,35E-06
LGALS3	ENSG00000131981	14	galectin 3	1,589357824	3,009153755	0,723536038
LARS2	ENSG00000011376	3	leucyl-tRNA synthetase 2, mitochondrial	1,580389838	2,990506469	0,025849185
SCML1	ENSG00000047634	X	Scm polycomb group protein like 1	2,85086263	7,214316067	3,52E-09
NBPF20	ENSG00000162825	1	Neuroblastoma Breakpoint Family, Member 20	3,029199621	8,163566761	0,013076519
TOR1AIP2	ENSG00000169905	1	torsin 1A interacting protein 2	3,84681594	14,38821722	6,11E-19
PPP1R2	ENSG00000184203	3	protein phosphatase 1 regulatory inhibitor subunit 2	4,073227898	16,83308732	1,35E-07

### ABX464 upregulates miR-124

**Figure 3:**
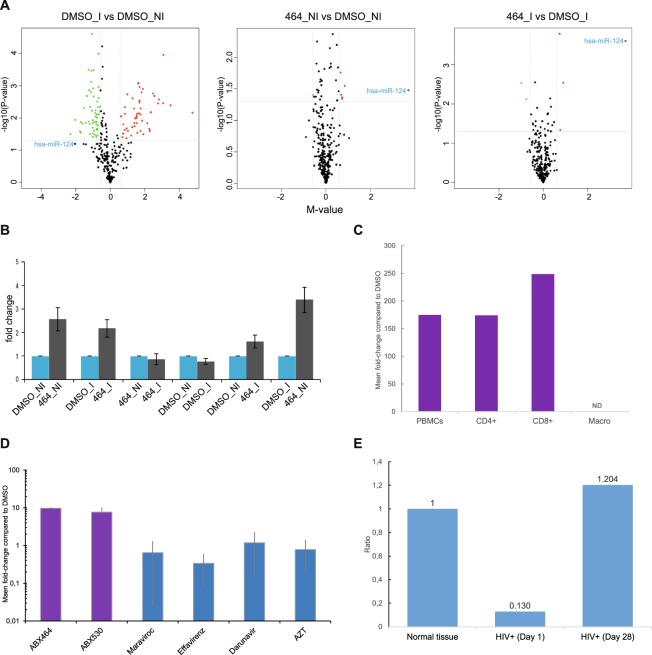
ABX464 upregulates a single microRNA, the anti-inflammatory miR-124. (**A**) Microarray analysis of small RNAs from PBMCs from 6 donors. PBMC were infected with the YU-2 strain (I) or uninfected (NI) and treated with ABX464 or untreated (DMSO). Volcano plots show that infection leads to large variations in small noncoding RNAs (left panel), whereas ABX464 induced a reproducible upregulation of a single microRNA, miR-124, in infected and uninfected cells (right and middle panels, respectively). (**B**) Quantification of miR-124 expression using TaqMan Low Density Array technology in CD4+ T cells under the same conditions as in A. (**C**) Expression of miR-124 measured by qPCR in PBMCs, purified CD4+ and CD8 T cells, and macrophages treated with ABX464 in comparison to untreated cells (DMSO, fold change). (**D**) Expression of miR-124 in PBMCs treated with the antiretrovirals ABX464, ABX530, maraviroc, efavirenz, darunavir and AZT compared to untreated cells (DMSO, fold change). (**E**) Quantification of miR-124 in rectal biopsies of healthy participants (n = 10) and HIV patients undergoing ART at days 1 and 28 of treatment with ABX464 (n = 9). Individual graphs show the results for each patient in comparison with the results for healthy participants.

Since the CBC complex, which is targeted by ABX464, is involved in the biogenesis of small noncoding RNAs, and our global analysis thus far did not include these, we decided to evaluate if miRNAs or small nucleolar RNAs (snoRNAs) were differentially regulated by ABX464. We performed a microarray analysis for these RNAs from the PBMCs of 6 donors. Cells that were infected with the YU-2 strain, followed by treatment with ABX464 were compared with uninfected and untreated controls. A total of 104 human miRNAs and 40 snoRNAs were significantly differentially expressed in infected PBMCs, when compared to uninfected PBMCs (data file S2), with a false discovery rate lower than 0.05 and fold change higher than 1.5. The cluster analyses revealed complete separation of the infected and uninfected samples based on the expression profiles of the differentially expressed miRNAs (Fig. 3A). While infection led to this substantial variation in the expression of small noncoding RNAs (Fig. 3A, left panel), ABX464 treatment induced reproducible upregulation of a single microRNA, miR-124, in infected and uninfected cells (Fig. 3A, middle and right panels respectively, data files S3 and S4). To confirm this result, we used another method of profiling microRNA, the TaqMan Low Density Array (TLDA), which is based on reverse transcription real-time PCR, with a screening capacity for 760 miRNAs (Fig. S4A). Again, only miR-124 was observed to be upregulated by ABX464. Furthermore, quantitative PCR of total RNA isolated from infected and uninfected PBMCs of 5 donors demonstrated that infection led to a slight reduction in miR-124 expression (Fig. 3B, also visible in the total analysis in Fig. 3A), but treatment with ABX464 resulted in significant upregulation of miR-124 in both infected and uninfected cells (Fig. 3B). Next, we determined whether the observed miR-124 response to ABX464 treatment in PBMCs could be attributed to specific cell types. Using purified CD4+, CD8 and macrophages cells, we found that ABX464 treatment resulted in upregulation of miR-124 in lymphoid cells (Fig. 3C) but not in monocyte-derived macrophages, where the expression of miR-124 was undetectable (Figs 3C and S4B). Finally, we proved that this upregulation of miR-124 is specific to the ABX class of small molecules, since other antiretrovirals such as maraviroc, efavirenz, darunavir and AZT did not upregulate the expression of miR-124 in PBMCs, whereas ABX530, a molecule that has the same properties as ABX464, induced upregulation of miR-124 to a similar extent as ABX464. (Fig. 3D).

In recent years, miR-124 has emerged as a critical modulator of immunity and inflammation. miR-124 is known both as a key regulator of microglia quiescence in the central nervous system and as a modulator of monocyte and macrophage activation^43,44^. miR-124 also plays a critical role in both innate and adaptive immune response^45–48^. In particular, miR-124 was shown to be a critical mediator of cholinergic anti-inflammatory action by reducing IL-6, TNF-α and MCP-1 production^49^. Interestingly, IL-6, TNF-α and MCP-1 were upregulated in the commonly used DSS-induced experimental mouse model of colitis, whereas ABX464 reduced the expression of these proinflammatory cytokines^1^. Persistent immune activation and systemic inflammation also play central roles in the pathogenesis of HIV disease^50,51^. Many HIV-infected individuals treated with antiretroviral therapy (ART) exhibit residual inflammation associated with non-AIDS-related morbidity and mortality^52–54^. Based on the observed downregulation of miR-124 expression upon HIV infection of PBMCs or CD4+ T cells, and subsequent upregulation with ABX464 treatment, we decided to examine if similar modulation of miR-124 expression was seen in HIV patients undergoing antiretroviral therapy (ART). Since the chronic inflammatory state in patients receiving combination ART is primarily related to the extent of damaged gut-associated lymphoid tissue^54–56^, miR-124 expression was monitored in rectal biopsies from HIV-infected patients undergoing ART and treated with ABX464 (N = 9). miR-124 expression was downregulated in HIV patients treated with ART, in comparison to its expression in colon biopsies from healthy donors (N = 10). Treatment with ABX464 for 28 days restored the expression of miR-124 to the level of healthy donors (Fig. 3E). When ABX464 treatment was stopped for 28 days, the expression of miR-124 decreased to reach the level seen before treatment (Fig. S4C,D), indicating that the changes observed in expression were specifically due to ABX464. Thus, ABX464 modulates the expression of miR-124 both *in vitro* and in HIV patients.

### ABX464-induced splicing of a long noncoding RNA at the miR-124-1 locus results in upregulation of miR-124

**Figure 4:**
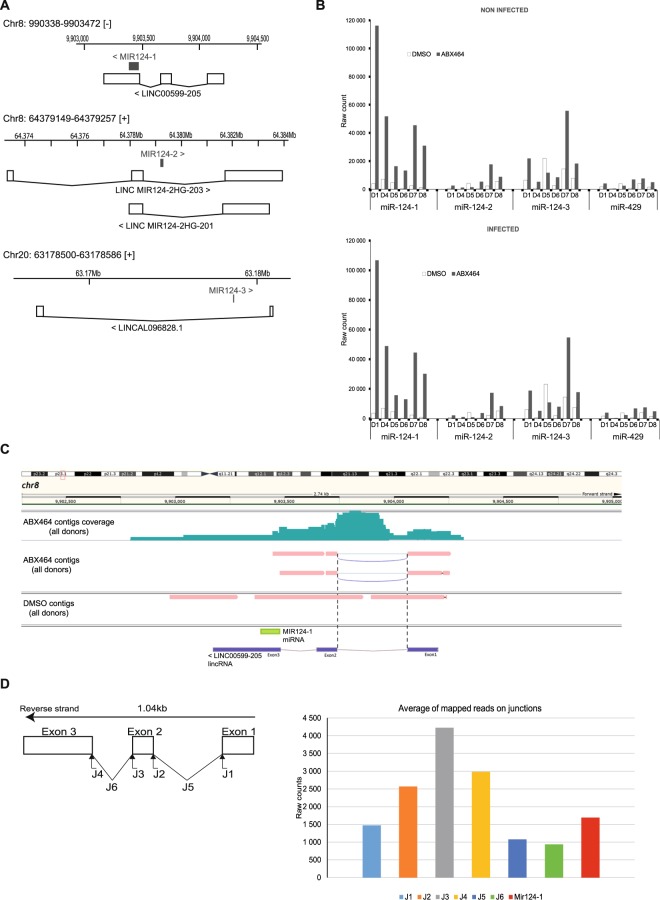
miR-124 upregulation by ABX464 originates from splicing of a long noncoding RNA at the *miR-124-1* locus. (**A**) There are three genes encoding miR-124, *miR-124-1*, *miR-124-2*, and *miR-124-3*, located on chromosomes 8 and 20 in the human genome. (**B**) We employed a targeted RNA capture and sequencing strategy to determine which gene was induced by ABX464. In both infected and uninfected cells, treatment with ABX464 leads to upregulation of miR-124 from the miR.124.1 locus, whereas a control locus, miR-429, is not affected. (**C**) Locus *miR-124-1* contains a long noncoding RNA (LncRNA 0599-205) whose splicing is stimulated by ABX464. (**D**) Counts of the reads at splice junctions (J1, J2, J3 and J4), exon-exon (J5 and J6) and the miR-124 75 bp region (miR-124) quantified by RNA CaptureSeq in PBMCs treated with ABX464.

miR-124 is encoded from three independent genes, *miR-124-1*, *miR-124-2*, and *miR-124-3*, located on human chromosomes 8 and 20 (Fig. 4A). To determine which of these genes is induced by ABX464, we employed targeted RNA CaptureSeq to assess the full depth of the transcriptome. The increased sequencing depth of RNA CaptureSeq together with *ab initio* transcript assembly was employed to determine which locus is affected by ABX464 treatment (Fig. 4A,B). We reconstructed all transcripts assembled within the precapture RNA-Seq data with a similar uniformity of transcript coverage (100% of transcript chains reconstructed; Fig. 4A,B). The total number of reads for untreated as well as ABX464 treated samples varied between 2 and 30 million in infected and uninfected PBMCs (Table S3). The number of reads from the three loci encoding miR-124 in the treated samples were 4- to 12-fold higher than in the untreated samples (Fig. 4B, Table S3), confirming that ABX464 led to a large increase in miR-124. In contrast, ABX464 had no effect on the expression of miR-429 (Fig. 4B), located outside the miR-124 region, again showing the specificity of ABX464 in targeting miR-124.

Most of the aligned reads from the 10-kb regions of each locus originated from *miR-124-1* and *miR-124-3*, whereas the number of aligned reads from *miR-124-2* was quite low even after ABX464 treatment (Fig. 4B). The effect of ABX464 on the expression of miR-124 was most pronounced from the *miR-124-1* locus (Fig. 4B). All mapped reads from the *miR-124-1* locus in the treated samples aligned to miR-124 and a 2 kb region surrounding it. Close inspection of this region indicated that the miR-124 sequence is embedded in a long noncoding RNA (lncRNA 0599-205) at the *miR-124-1* locus (Fig. 4A,C).

The sequencing depth of RNA CaptureSeq permitted us to assemble *ab initio* transcripts exhibiting a complex array of splicing patterns. This approach revealed that splicing of lncRNA 0599-205 is activated by ABX464, and this splicing was not present in untreated samples (Fig. 4C,D). Most of the contigs in the untreated samples aligned with unspliced lncRNA 0599-205. Mapping the reads at the splice junctions (J1, J2, J3 and J4) and exon-exon junctions (J5 and J6) of this lncRNA allowed quantification of spliced and unspliced RNA in the treated samples (Fig. 4D). After 8 days of treatment with ABX464, which we assumed would be enough time for the transcripts to reach steady-state levels, the level of unspliced RNA was nine times higher than that of spliced RNA. Since spliced RNA is more stable than unspliced RNA, we must consider that spliced lncRNA 0599-205 is a source of miR-124 upregulation and therefore becomes less stable. Consistent with this prediction, we found that miR-124 production (reads that mapped to only the 85 bp miR-124 region) compensated for the lack of spliced lncRNA 0599-205 (Fig. 4D).

### Splicing of lncRNA 0599-205 is required for the production of miR-124

**Figure 5:**
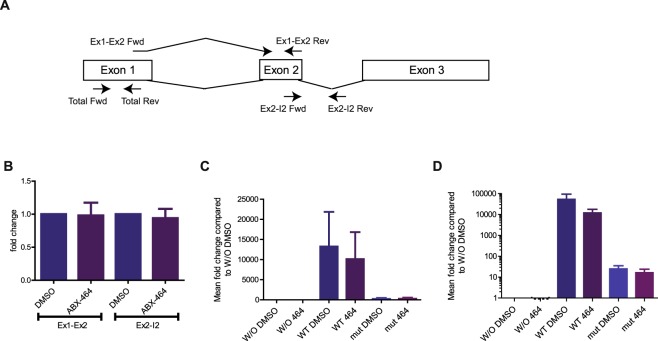
Splicing of LncRNA 0599-205 is required for the production of miR-124. (**A**) Schematic representation of LncRNA 0599-205 precursor and primers used to amplify different derived RNAs. (**B**) Quantification of spliced and unspliced LncRNA 0599-205 in the presence or absence of ABX464. (**C**) Quantification of the expression of miR-124 following transfection of a wild type and splicing mutant of lncRNA0599-205 plasmids in HeLa cells in the presence or absence of ABX464. (**D**) Quantification of total wild type and splicing mutant lncRNA0599-205 in the presence or absence of ABX464.

To directly assess the contribution of lncRNA 0599-205 to the production of miR-124, we cloned the genomic sequence of chromosome 8 from 9 903 167 through 9 904 210 of lncRNA 0599-205 into a plasmid vector (Fig. 5A). Both the spliced and unspliced transcripts of lncRNA 0599-205 could be readily detected in the transfected HeLa cells (Fig. 5B). However, we failed to detect an upregulation of miR-124 in transfected HeLa cells in response to ABX464 treatment since splicing is maximized in HeLa cells and the CBC complex would have an effect at the site of transcription (Fig. 5B). However, transient transfection of the plasmid into HeLa cells resulted in a dramatic (1 250-fold) increase in miR-124 levels (Fig. 5C, left panel). In contrast, when the splice sites of the lncRNA 0599-205 are mutated only traces of miR-124 are detected (Fig. 5C, left panel). Consistent with the fact that unpliced lncRNA 0599-205 are less stable, the amount of mutated lncRNA 0599-205 is less than wild type (Fig. 5C, right panel). All together, the results demonstrate that splicing of the lncRNA 0599-205 is a prerequisite for the production of mir-124.

## Discussion

Our investigation provides new insights into the mechanism of action of ABX464 in stopping HIV infection and upregulating the anti-inflammatory miR-124 in infected patients undergoing ART treatment. ABX464 binds CBC (ABIVAX, data in file), a complex that stimulates processing reactions of capped RNAs, including their splicing, 3′-end formation, degradation, and transport. Given that the CBC is believed to bind all classes of m7G-capped RNAs, including precursors and mature forms of mRNAs, stable long noncoding RNAs (lncRNAs), nonadenylated histone RNAs, and precursors of spliceosomal small nuclear RNAs (snRNAs), it is important to understand how the binding of ABX464 to CBC complex achieves its specificity. Both the variation of splicing of HIV RNAs and lncRNA 0599-205 are robustly induced by ABX464. We don’t know the reason for that. It could be that weak splice sites that characterizes both transcripts, is at the origin of the induction of these splicing events by ABX464-CBC complex. Before to be exported to cytoplasm most of human protein coding genes are spliced, if not they are degraded in the nucleus, and in the cytoplasm a pioneer round of translation is important for mRNA quality control. The lncRNA 0599-205 like any RNA in the cell will be degraded or stay at its site of transcription which is the case of many long coding RNA. The miR-124 sequence is at the 3^rd^ exon of lncRNA 0599-205, this is also a peculiar situation because most of microRNA are generally classified as “intergenic” or “intronic” based upon their genomic location. Intergenic miRNAs are known to be transcribed as independent transcription units, while intronic miRNAs are believed to be processed from the introns of their hosting transcription units and hence share common regulatory mechanisms and expression patterns with their host genes. Since ABX464 treatment did not induce any variation of the microRNA other than miR-124 and very little variation of expression of genes that might host microRNA, we believe that ABX464-CBC interaction induces a specific effect of viral RNA and miR-124 biogenesis. This latter point is also demonstrated by the fact that miR-124 is transcribed by 3 loci but only the miR-124.1 locus is affected by ABX464 treatment and also the splicing of lncRNA 0599-205 is required for the production of miR-124 (Fig. 5). In keeping with this, the mouse miR-124.1 locus also harbor a lncRNA (Rncr3) which serves as pri-miR124 to generate miR-124 and this expression is modulated at post-transcriptional level by the splcing factor PTB1 which inhibits the production of miR-124 by binding the Rnc3 RNA^57^.

Nuclear cap functions are mediated by the CBP80 and CBP20 proteins, which associate cotranscriptionally with the nascent RNA^8,58,59^. CBP20 interacts directly with the m7G cap through its classical RNA recognition motif (RRM), while CBP80 ensures high-affinity binding of the full CBC and provides a platform for interactions with other factors^4,60,61^. We have demonstrated that ABX464, by binding the CBC, prevents the production of the unspliced viral RNA required for viral replication and induces the production of miR-124 from lncRNA-0599-205. However, ABX464 had a negligible impact on cellular splicing. Therefore, ABX464 acts as an enhancer of viral but not cellular splicing. Binding of ABX464 may change the conformation of the CBC to allow efficient interaction of CBP80 with a splicing factor and thereby render recognition of the viral splicing sites more efficient. It is known that HIV RNA has suboptimal splicing sites to allow inhibition of splicing of the 9 kb primary transcript at later stages of infection^18,62^. In contrast, most cellular genes require splicing for expression, and unspliced intron-containing transcripts are retained in the nucleus where they are degraded^20^. Depletion of NCBP1 by 50% leads to upregulation of unspliced IR transcripts confirming the implication of CBC in NMD. ABX464 is making sure that viral HIV RNA is spliced (since it is transcribed from integrated pro viral DNA resembling any gene in the cell), exported and unspliced viral RNA is degraded (presumably by NMD). ABX464 is not changing any of CBC functions therefore it is not expected that ABX464 by binding the CBC complex change the general RNA biogenesis in the cell. However, very little is known about CBC complex under HIV infection or immune cells activation. The finding that ABX464 binds to CBC and induce specific changes in immune cells; miR-124 overexpression by modulating the splicing 0599-205 long coding RNA and enhancement of splicing of HIV RNA, will be of great help to understand the involvement of CBC under HIV infection and inflammation.

A prediction of splice site strength using maximum entropy (MaxEnt) revealed that the 5′ splice site between exon 2 and intron 2 of lncRNA 0599-205 is very weak compared to the other splice site (Table S4). Since unspliced lncRNA 0599-205 cannot exit the nucleus due to quality control machinery and CBC-mediated control, spliced lncRNA 0599-205 constitutes a miRNA storage form, possibly in addition to other functional properties of the intact spliced transcript. This storage may be maintained through low transcriptional and degradative activity of unspliced lncRNA 0599-205 and producing only low levels of mature miR-124 release under normal conditions. Indeed, when lncRNA 0599-205 is unspliced, its fate is similar to that of other short-lived transcripts^63^, susceptible to a CBC complex called CBC-NEXT^64^, which promotes RNA degradation via the nuclear RNA exosome^65^. Consistently, the splicing of lncRNA 0599-205 is a prerequisite for the production of miR-124. The splicing of lncRNA 0599-205 is important to stabilize the mature transcript for recognition by the microRNA processing machinery and thereby produce miR-124. ABX464 treatment could, thus, enable the rapid release of a large amount of miR-124 through lncRNA 0599-205 splicing without requiring transcriptional activation of the lncRNA 0599-205 locus. In HIV patients this could be the mechanism by which miR-124 is up-regulated following treatment with ABX464 (Fig. 3E).

HeLa cells, which contain a large excess of splicing factors, efficiently produce HIV viral RNA splice products^66^ and a large amount of miR-124 from transfected HIV and lncRNA 0599-205 constructs, respectively. Thus, ABX464 does not impede the normal function of CBC in splicing but renders it more efficient. As viral but not cellular genes require the unspliced state for viral replication, and splicing of lncRNA 0599-205 can affect miR-124 expression, ABX464 inhibits viral replication and induces anti-inflammatory miR-124 expression.

Large numbers of studies in different cells and systems have found that miR-124 is a critical modulator of inflammatory and immunological responses. The finding that ABX464 upregulates miR-124 expression in infected patients could be a major advantage in treating residual inflammation associated with non-AIDS-related morbidity and mortality in HIV patients^52–54^. In many cases, miR-124 functions as a negative regulator for inflammatory signals, providing negative feedback to help maintain homeostasis. Furthermore, Ulcerative colitis patients treated with ABX464 also demonstrate elevation of miR-124 and show evidence of a robust and consistent efficacy signal of ABX464 (Unpublished results). Thus, ABX464 is a small molecule with therapeutic potential not only against HIV, but also against inflammatory diseases.

## Materials and Methods

### Human samples

All experiments on human biopsies were performed in accordance with relevant guidelines and regulations. The experiments were approved by the AEMPS (Spanish Agency) **N° AEMPS:** 16-0728 under the contract N° **EudraCT:** 2016-002797-1título ESTUDIO ABIERTODE LA SEGURIDAD, FARMACOCINÉTICA Y FARMACODINÁMICA DE ABX464 EN ADULTOS SERONEGATIVOS Y SEROPOSITIVOS PARA EL VIH-1. Informed consent was obtained from all subjects.

### Cell culture, infection and transfection

Buffy coats from HIV-negative individuals were obtained from the local blood donation center in Centre de transfusion sanguine Montpellier. Human peripheral blood mononuclear cells (PBMCs) were isolated by Ficoll (Histopaque, Sigma) gradient centrifugation. CD4+ and CD8+ T cells were purified from PBMCs after Ficoll gradient centrifugation by Human CD4+ beads positive selection and CD8+ beads positive selection, respectively (Miltenyi). PBMCs, CD4+ and CD8+ T cells were grown at 37 °C and 5% CO_2_ to a density of 1.5 × 10^6^ cells/ml in RPMI GlutaMAX medium (Life Technologies) supplemented with 10% fetal calf serum (FCS) (Thermo Fisher), 40 U/ml of IL2 (PeproTech) and 5 µg/mL of PHA (Roche) for 2 days. Three days later, cells were resuspended at 1.5 × 10^6^ cells/ml in RPMI supplemented with 10% FCS and 40 U/ml IL2 and separated into two parts (infected and uninfected cells). Cells were infected with 80 ng of p24/10^6^ cells of the YU-2 strain for 4 to 6 hours and then rinsed with PBS before medium renewal.

Both infected and uninfected cells were then centrifuged and resuspended to a density of 1.5 × 10^6^ cells/ml in medium supplemented with diluted drug solubilized in DMSO (Sigma) to a final 0.05% DMSO concentration or antiretroviral compounds according to the manufacturer’s instructions. Cells were treated for 6 days with a medium renewal at day 3. HIV p24 titration was performed by ELISA from cell culture supernatants with Innotest kit (Ingen) according to the manufacturer’s instructions.

To generate monocyte-derived macrophages, monocytes were isolated using CD14+ positive microbeads (Miltenyi) and cultured in X-VIVO 10 medium (Lonza) supplemented with 10 mM HEPES, 1 mM sodium pyruvate, 1% non-essential amino acids, 10% FBS, 10 ng/ml GM-CSF and 100 ng/ml M-CSF for 7 days with medium renewal at day 3. After 7 days of differentiation, the cells were washed, plated in 6-well plates and incubated for 24 hours in medium without cytokine addition. Cells were treated for 6 days with a partial medium change at day 3.

HeLa cells from ATCC were cultivated at 37 °C and 5% CO_2_ in DMEM GlutaMAX medium (Life Technologies) supplemented with 10% FCS (Thermo Fisher). The region corresponding to lncRNA0599-205 was synthesized by IDT (Integratde DNA Technologies) and cloned into pcDNA3.1 to generate the pc-lncRNA-0599-205 plasmid. pc-lncRNA-0599-205 (2 µg per 400,000 cells) was transiently transfected in HeLa cells using jetPEI reagent (Polyplus) according to the manufacturer’s instructions.

RNA CaptureSeq of HIV RNA and cellular microRNAs, sequencing and bioinformatics analysis, are included as Supplementary Materials.

### miRNA (RT-q)-PCR analysis

miRNAs were extracted from PBMCs, CD4+ or CD8+ T cells using the NucleoSpin miRNeasy kit (Macherey-Nagel) according to the manufacturer’s instructions. miRNA reverse transcription (RT) was performed using the miScript II RT kit (Qiagen). The resultant cDNA was used as a template for real-time qPCR performed with the miScript SYBR Green PCR kit (Qiagen), together with the appropriate primers (Qiagen). A specific miR-124 primer (Hs_miR-124a), a control-specific primer (Ce_ miR-39) and two housekeeping gene (miR-26 and miR-191)-specific primers (Hs_miR-26a and Hs_miR-191) were used to perform relative qPCR. The PCR analysis was performed on a LightCycler 480 Instrument II (Roche Molecular Systems, Inc). All reactions were run in triplicate. The relative levels of miR-124 were calculated using the 2^−ΔΔCt^ method.

Total RNA was extracted from macrophages using the Macherey-Nagel NucleoSpin miRNeasy kit. miRNA reverse transcription (RT) was performed on 2 µl of total RNA according to the Applied Biosystems TaqMan Advanced miRNA assays protocol for TaqMan qPCR. A specific miR-124 primer (Hs_miR-124a), a spike control-specific primer (Ce_miR-39) and housekeeping gene (miR-191)-specific primers (Hs_miR-191) were used to perform relative qPCR. PCR runs were analyzed with Applied Biosystems ViiA 7 software.

### miR-124 quantification in human biopsies

Quantification of miR-124 was performed on rectal biopsies sample from patients that have been collected in PAXgene Tissue Containers (PreAnalytiX). RNA extractions were performed using the PAXgene Tissue miRNA Kit (PreAnalytiX) following manufacturer’s protocol. Briefly, samples were immersed in 250 µl of Buffer TM1 in a 2 ml Safe-Lock microcentrifuge tube containing one 5 mm stainless bead. Samples were disrupted and homogenized using Qiagen TissueLyser apparatus during 2 × 2 minutes at 20 Hz. The rest of the procedure was performed following the manufacturer’s protocol. At the end of the procedure, each RNA was eluted into 32 µl of TM4 Buffer. RNA concentration and purity were measured using a NanoDrop ND-1000 spectrophotometer (Thermo Scientific). RNA integrity was assessed using an Agilent 2200 TapeStation with RNA ScreenTape. cDNA templates were prepared using the TaqMan Advanced miRNA cDNA Synthesis Kit (Applied Biosystems) starting from the 10 ng total RNA matrix and following the manufacturer’s protocol. qPCR was carried out using two TaqMan Advanced miRNA Assays (Applied Biosystems), one targeting the miR-124-1 miRNA (assay ID: 477879_mir) and the other targeting an endogenous miRNA, miR16 (assay ID: 477860_mir), which was used as a reference for expression data normalization, according to the manufacturer’s instructions. QPCR was performed on the LightCycler 480 Instrument II (Roche Molecular Systems, Inc). All assays were labeled using FAM-MGB chemistry.

### Statistical data analysis

All statistical analyses and plots were done with R software v3.5. For normalization and processing, we used edgeR v3.22.1, EDASeq v2.14 and DESeq2 v1.20. We decided to use counts per million (CPM) over transcripts per million (TPM) mainly because TPM discards a lot of information about the original count sizes and thus would have produced too much noise for differential testing. The first step was to filter the data. We considered a gene to be expressed at a reasonable level in a sample if it had at least five counts for each million mapped reads in that sample. For marker selection, we used a type 1(*) error α threshold set to 5%, enabling a highly flexible approach to the differential analyses despite the lack of replicates. For the same reason, the log_2_ fold change cutoff was set to 1.5 for both positive and negative values, and the cutoff *p*-value was typically ≤0.05. Those appropriate cutoffs allowed us to to identify differentially expressed genes. The false discovery rate (FDR) values were computed for illustration purposes but were not taken into account in the decision due to the lack of replicates. The first methodology choice was Benjamini and Hochberg. This procedure provides less stringent control of type 1 errors compared to familywise error rate (FWER) procedures (such as the Bonferroni correction). We added an MDS plot for the data with R and edgeR package. The MDS plot produces a plot in which distances between samples correspond to the leading biological coefficient of variation (BCV) between those samples. In this plot, we did not observe segregation between the DMSO-NI, 464-NI, DMSOI and 464-I conditions. We also produced several volcano plots with R and the EDASeq package. These plots were a good representation of the differential expression (DE) testing in the RNA-Seq experiments. These plots showed the log_2_ fold-change (FC) and the –log_10_ of *p*-values for all genes investigated in the DE test. The green points highlighted are markers that satisfied our selection criteria (significant in both *p*-value and log_2_FC). The yellow points highlighted are genes that satisfied the log_2_FC criterion but failed the *p*-value criterion. We retained these points to investigate further even though they had no decisional value.

## Supplementary information

Supplementary information

supplementary data file S1

supplementary data file S2

supplementary data file S3

supplementary data file S4

## Data Availability

The deep sequencing data are available through https://www.ncbi.nlm.nih.gov/geo/query/acc.cgi?acc=GSE116073 and microarray data through. ABX464 must be obtained through an MTA.
