# Supplementary material for: Both anti-inflammatory and antiviral properties of novel drug candidate ABX464 are mediated by modulation of RNA splicing
Source: Sci Rep. 2019 Jan 28;9:792. doi: 10.1038/s41598-018-37813-y (PMC6349857; doi:10.1038/s41598-018-37813-y)
Supplement: Supplementary file 1 — Supplementary information [file 41598_2018_37813_MOESM1_ESM.pdf]

# Both anti-inflammatory and antiviral properties of novel drug candidate ABX464 are mediated by modulation of RNA splicing

Audrey Vautrin[2] †, Laurent Manchon[1] †, Aude Garcel [2], Noëlie Campos[2], Laure Lapasset[2], Mahdi Laaref[1], Roman Bruno[3], Marie Gislard[4], Emeric Dubois[4], Didier Scherrer[2], Hartmut Ehrlich[2] and Jamal Tazi[1*]

[1] IGMM, CNRS, Univ. Montpellier, Montpellier, France
1919 route de Mende, 34293 Montpellier Cedex 5, Montpellier, France
[2] ABIVAX, 1919 route de Mende, 34293 Montpellier Cedex 5, France
[3] ACOBIOM, 1682 Rue de la Valsière, 34184 Montpellier Cedex 4, France
[4] MGX, Univ Montpellier, CNRS, INSERM, Montpellier, France
† These authors contributed equally to this work

Supplementary Materials and Methods

Sequencing and bioinformatics analysis

RNA CaptureSeq of HIV RNA and cellular microRNAs

Total RNA was extracted from infected and uninfected PBMC samples using TRIzol reagent (Invitrogen) following the manufacturer's protocol. The RNA concentrations and purity were determined using the RNA 6000 Bioanalyzer kit (Agilent). For cDNA synthesis, 1 μg of total RNA was treated with DNase I (Invitrogen) and then utilized as a template for reverse transcription using the Verso cDNA kit with a blend of random hexamers and anchored oligo-dT (Thermo Scientific), according to the manufacturer's instructions. After KAPA Hyper Prep (ROCHE) library preparation from 1μg total RNA, we proceeded with SeqCap EZ capture (ROCHE) following the manufacturer's recommendations. Hybrid selection was performed with a custom SeqCap EZ Choice Library (Roche NimbleGen) containing the following regions; chromosome 8 (9,760,617 to 9,761,780 and 65,285,285 to 65,296,944), chromosome 20 (61,797,549 to 61,812,855), chromosome 1 (1,102,000 to 1,106,000), chromosome 13 (92,000,074 to 92,006,833), chromosome 15 (22,512,831 to 2,513,641) and the genome of the HIV Ada8 strain. This library was designed through the NimbleDesign portal (v1.2.R1) using genome build hg19 NCBI Build 37.1/GRCh37. Sequencing was performed on a NextSeq 500 (Illumina) with 18 samples on a HighOutput Cartridge (2x400 million reads of 75 bases each).

CD4+ T cells sequencing

A full-detailed report describing the complete analysis was submitted to Gene Expression Omnibus (GEO, https://www.ncbi.nlm.nih.gov/geo/query/acc.cgi?acc=GSE116073). Total RNA extraction

was performed according to the Qiagen miRNeasy® kit. RNA concentrations were determined using Qubit® fluorometric quantification and purity by the RNA 6000 Bioanalyzer kit (Agilent). RNA-Seq libraries were constructed with the TruSeq stranded mRNA sample preparation (low-throughput protocol) kit from Illumina, according to the manufacturer's instructions. Aliquots of 1.2 µg of total RNA were used for the construction of the libraries. The final cDNA libraries were validated with a Fragment Analyzer (Advanced Analytical, Ankeny, IA) and quantified with a KAPA qPCR kit (Kapa Biosystems, Wilmington, MA). On the 8 sequencing lanes of a flow cell V4, the 32 libraries were pooled in equal proportions by sets of 4 libraries per lane, denatured with NaOH and diluted to 16 pM before clustering. Cluster formation and primer hybridization and single-end read, 50-cycle sequencing were performed on the cBot and HiSeq 2500 (Illumina, San Diego, CA), respectively. Image analysis and base calling were performed using the HiSeq Control Software with the Real-Time Analysis component. Demultiplexing was performed using Illumina's sequencing analysis software. The quality of the data was assessed using FastQC from the Babraham Institute and the Illumina software SAV (Sequence Analysis Viewer). Potential contaminants were investigated with the FastQ Screen software from the Babraham Institute.

To investigate splicing activity we used SUPPA v2.0.0, a tool developed in Python 3.4 and especially designed for this approach. To study splicing across multiple conditions, we performed three modular operations that were run separately. The first step was to generate events from the annotation file commonly named the GTF file (general transfer format). The second step was to quantify event inclusion levels (PSIs) from our samples. Finally, differential splicing across multiple conditions was calculated for all replicates.

To generate the different alternative splicing events from an input annotation file (GTF format), the method reads transcript and gene information solely from the "exon" lines

in the GTF. It then generates the events and outputs an 'ioe' file, which contains the relationship between each event and the transcripts that share this particular event. Specifically, it provides the transcripts that contribute to the numerator (one form of the event) and the denominator (both forms of the event) of the PSI calculation. For the generation of PSI values, SUPPA reads the ioe file generated in the first step and a transcript expression file with the transcript abundances.

SUPPA calculates the magnitude of the splicing changes (ΔPSI) and their significance across the biological conditions, using four replicates per condition. Conditions are analyzed in a sequential order specified as input. Statistical significance is calculated by comparing the observed ΔPSI between conditions with the distribution of the ΔPSI between replicates as a function of gene expression (measured as the expression of the transcripts defining the events). Using the output from the differential splicing analysis, we classified the events into four major groups: Alternative 5' splice site (A5), Alternative 3' splice site (A3), Alternative first and last exons (Altexons) and Retained intron (RI). Using this classification, we classified the differential splicing events encountered in the different comparisons of samples using a very stringent cutoff of psi-score difference (ΔPSI) fixed at > 0.4 and a p-value < 0.05. We used simple Perl scripts to establish raw count tables from the SUPPA outputs.

MicroRNA array analysis

Total RNAs were extracted from infected and uninfected PBMC samples using miRNeasy kit (Qiagen) following the manufacturer's protocol. The RNA concentrations were determined using the Nanodrop spectrophotometer (ThermoFisher). Quality controls were performed using expression console metrics. A full-detailed report describing the complete analysis was submitted to GEO (https://www.ncbi.nlm.nih.gov/geo/query/acc.cgi?acc=GSE116148). All miRNA arrays were normalized using "RMA + DABG" normalization method within the Affymetrix Expression

Console with dedicated annotation files downloaded from the Affymetrix web server. Probes were considered expressed if the DABG p-value was less than 0.05 and not expressed otherwise. A miRNA was considered expressed in one condition if the probe was expressed in ¾ or more of the replicates. Finally, a miRNA was considered differentially expressed between conditions A and B if 1) it was expressed in A or B; 2) its fold change was ≥ 1.5; and 3) its paired t-test p-value was ≤ 0.05.

The volcano plots were produced using R. They show the M-value ($\log_2$(fold change)) and the -$\log_{10}$(p-value) of all miRNAs for the comparisons 464_I vs. DMSO_I, 464_NI vs. DMSO_NI and DMSO_I vs. DMSO_NI. The significantly (FC ≥ 1.5 and p-value ≤ 0.05) upregulated miRNAs were highlighted in red and the downregulated miRNAs in green.

TaqMan Low Density Array (TLDA)

MicroRNA profiling of samples was performed using TaqMan Array Human MicroRNA panels A and B (Life Technologies). Each TLDA card detects 384 features, including 377 human miRNAs, three endogenous small RNA controls (one of them being in quadruplicate), and a negative control. In total, 754 human miRNAs were quantified. Reverse transcription and preamplification were performed following the manufacturer's instructions (Megaplex™ RT Primers and Megaplex™ PreAmp Primers, Life Technologies). Nine microliters of diluted preamplified product added to 900 µL of total mix were used per TLDA card. Real-time quantitative PCR was performed with the ViiA 7 real-time PCR system, and the data were collected with the manufacturer's ViiA™ Software. Gene Expression Suite software (Applied Biosystems) was further used to process the array data. Automatic thresholds were checked individually and corrected when necessary.

In the data preprocessing step, the HiSeq control and real-time analysis software generates image analysis, base calling, and base call quality automatically in real time. "Dirty" raw reads were defined as reads that contained adaptor sequence or high content of unknown

bases and low-quality reads. These reads were removed before downstream analysis to decrease data noise. The filtering steps were as follows:

The quality of the RNA-seq libraries was first evaluated using the FastQC v0.11.5 software. We use the FastQ Screen Contaminant finder to test the alignment of a large set of data to different genomes representing potential sources of contamination. The software generates a graph showing the proportions of reads aligning with the different genomes tested. FastQ Screen uses the Bowtie2 aligner. Alignment is performed on a subset of the sequences of each sample.

Then, the reads were subjected to standard quality control (QC) and filter criteria according to the following parameters: (1) trimming and cleaning reads that aligned to primers and/or adaptors, (2) reads with over 50% low-quality bases (quality value ≤15) in one read, and (3) reads with over 10% unknown bases (N bases). We used a program called Trimmomatic to remove primers and bad quality reads. After filtering, we removed short reads (<36 bp); the remaining reads are called "clean reads" and were stored in FASTQ format.

Once we were confident in the quality of our sequencing data, we proceeded to align the clean reads within FASTQ files to a reference sequence. Alignments were performed using the TopHat2 v2.0.8b tool against the human GRCh38 reference genome.

TopHat2 is a splice-aware aligner that uses the Bowtie2 aligner to align the data before counting the reads that are mapped to each gene. Bowtie2 behavior was adjusted to support local alignment by setting it to sensitive. TopHat2 outputs were stored in BAM files (accepted_hits.bam, typically) that contained all the alignments. TopHat2 outputs also reported alignment statistics. As the SAM file format was then required to count the reads, BAM files were sorted by name and converted to SAM format using the SAMtools-1.3.1 software. To compute gene level raw counts from the mapped reads in each library, we used the tool htseq-count v0.6.1p1 of the Python package HTSeq, using the default union-counting mode. The

HTSeq package produced a raw counts table for each library, which was needed to determine each gene's expression level. DEG (differentially expressed gene) screening aims to find genes differentially expressed between the samples and perform further functional analysis on them. Gene expression levels and DEGs were computed by R software and a suite of R packages. We opted to focus our efforts and limit our statistical analysis to only specific libraries to outline and gain a deeper understanding of the effect of ABX464. In our work, we decided to compare DMSO versus DMSOi libraries, ABX464 versus DMSO libraries and 464i versus DMSOi libraries. Additionally, to obtain a meaningful result, in our statistical design, we considered the 4 donors as biological replicates.

Flow cytometry

PBMCs or CD4+ T cells from different donors in suspension were labeled with appropriate anti-human monoclonal antibodies (mAb) (all from BioLegend). Washing and reagent dilutions were performed with FACS buffer (PBS containing 2% fetal calf serum and 0.05% sodium azide [NaN_3]). All acquisitions were performed on a Cyan ADP (Beckman Coulter) flow cytometer. Data were analyzed with FlowJo software (Ashland, OR). Cellular debris and dead cells were excluded by their light-scattering characteristics.

Measurement of unspliced and spliced lncRNA 599-205

The retrotranscription (RT) reactions were performed with 1 µg of total RNA using the Maxima First Strand cDNA Synthesis Kit (ThermoFisher Scientific). Genomic DNA elimination was performed before amplification, but nevertheless, an RT negative control was performed without enzyme to confirm the absence of DNA contamination. qPCR was performed with the LightCycler® 480 SYBR Green I Master Mix (Roche) and the following primers: total lncRNA 599-205 Fwd (CCCTCCACCACTTGGGAC) and total lncRNA 599-205 Rev (GACCTGGGGATTCAGCCTTC), unspliced lncRNA 599-205 Fwd (GAACAAAGAGCCTTTGGAAGAC) and unspliced lncRNA 599-205 Rev

(GGAAGGGACCACAGCATC), spliced lncRNA 599-205 Fwd (CACTCAGCGATGGAGGAAA) and spliced lncRNA 599-205 Rev (CCAATCACACAGACAATGAGATAAC), internal control β-actin Fwd (GTGAAGGTGACAGCAGTCGGTT) and β-actin Rev (GAAGTGGGGTGGCTTTTAGGA). The amplification run conditions were as follows: 95°C (10 seconds), 58°C (30 seconds), and 72°C (30 seconds) for 40 cycles.

Figure S1

A Processing steps and quality control of data

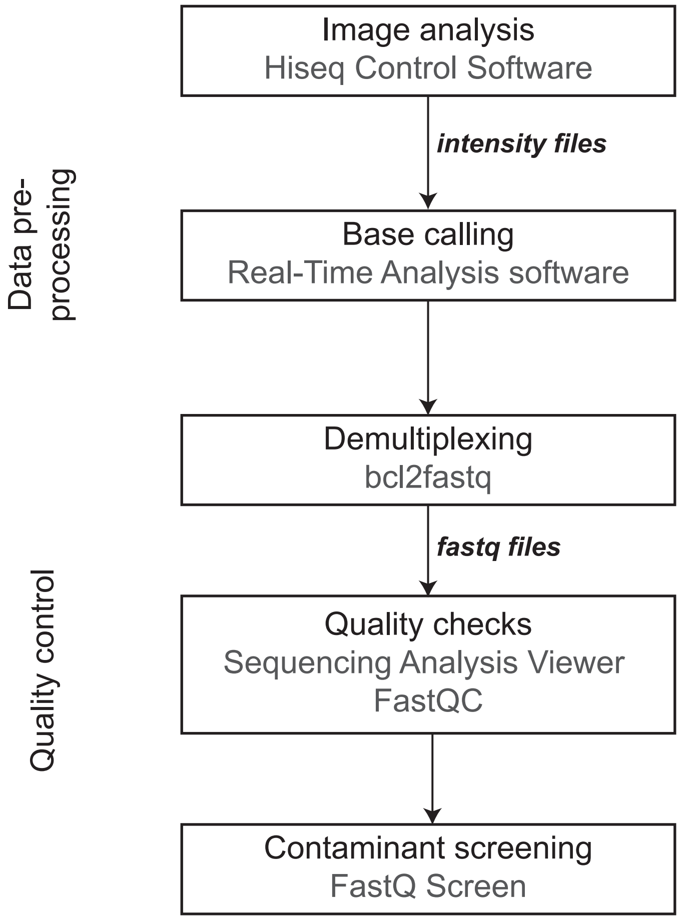

B The RNA-Seq processing pipeline

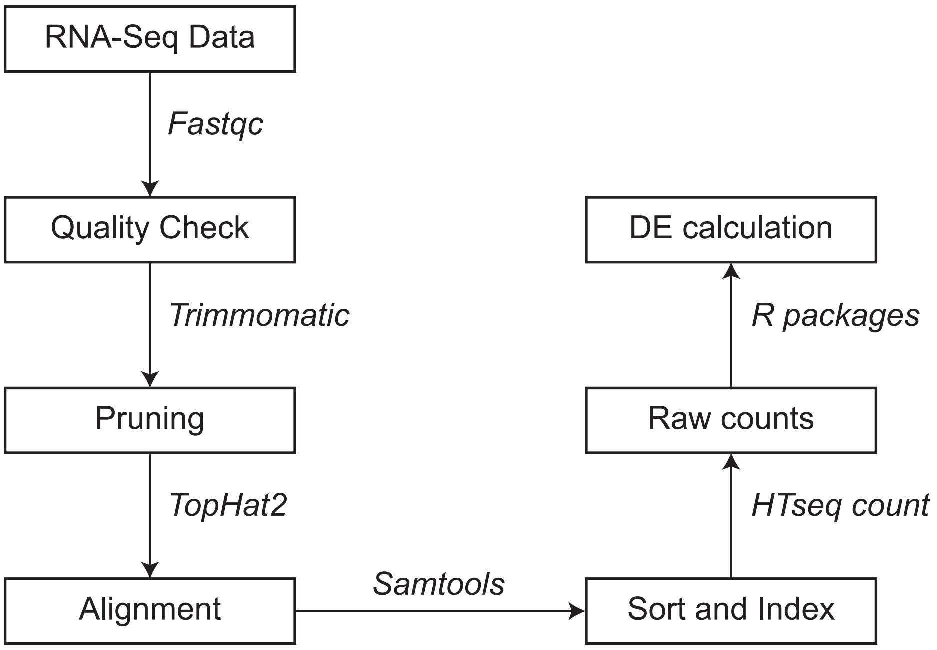

Supplementary Figure 1 : a. Processing steps and b. the RNA-seq processing pipeline.

Table S1

LIB names	Total paired-end reads before cleaning	Total paired-end reads after adapters cleaning	Total paired-end reads after contaminants cleaning	Total assembled contigs	Mapped contigs
D1_DMSO	7 948 802	7 884 109	7 006 433	683	63
D4_DMSO	30 625 849	30 363 868	27 810 800	1873	373
D5_DMSO	27 140 691	26 874 233	23 571 947	693	273
D6_DMSO	2 997 402	2 927 628	2 674 892	558	38
D7_DMSO	15 024 678	14 803 409	13 887 294	1489	190
D8_DMSO	17 570 815	17 386 693	16 491 699	1687	180
D1_464	4 940 644	4 879 559	4 557 902	362	90
D4_464	30 800 345	30 505 238	28 468 138	1413	225
D5_464	1 973 736	1 939 172	1 826 516	293	17
D6_464	4 450 825	4 385 103	4 174 706	227	49
D7_464	7 374 222	7 279 017	6 912 908	982	46
D8_464	8 507 861	8 381 026	7 835 264	759	48

Supplementary Table 1 : Read assemblies and contig counts from PBMCs infected with the YU2 strain, either untreated (D1_DMSO, D4_DMSO, D5_DMSO, D6_DMSO, D7_DMSO and D8_DMSO) or treated with ABX464 (D1_464, D4_464, D5_464, D6_464, D7_464 and D8_464).

Table S2

YU2 libraries	Complete_mRNA	Gag	Pol	Vif	vpr	tat	rev	vpu	env
D1_DMSO	123	6	9	2	6	2	2	5	3
D4_DMSO	174	6	12	5	5	7	9	11	8
D5_DMSO	194	6	7	6	4	8	11	5	10
D6_DMSO	80	3	10	3	2	8	2	4	2
D7_DMSO	146	6	6	2	7	2	8	3	6
D8_DMSO	166	8	4	9	2	4	3	10	4
D1_464	11	18	67	32	14	16	23	7	59
D4_464	13	42	61	34	14	18	21	5	42
D5_464	6	25	17	15	9	19	17	13	37
D6_464	32	13	14	16	12	15	9	11	13
D7_464	15	19	12	12	11	18	12	11	35
D8_464	6	26	35	25	12	24	29	17	42

Supplementary Table 2 : Contig counts of complete mRNA or spliced RNA transcripts of Gag, Pol, vif, vpr, tat, rev, vpu and env from PBMCs infected with YU2 strain, either untreated (D1_DMSO, D4_DMSO, D5_DMSO, D6_DMSO, D7_DMSO and D8_DMSO) or treated with ABX464 (D1_464, D4_464, D5_464, D6_464, D7_464 and D8_464).

Figure S2

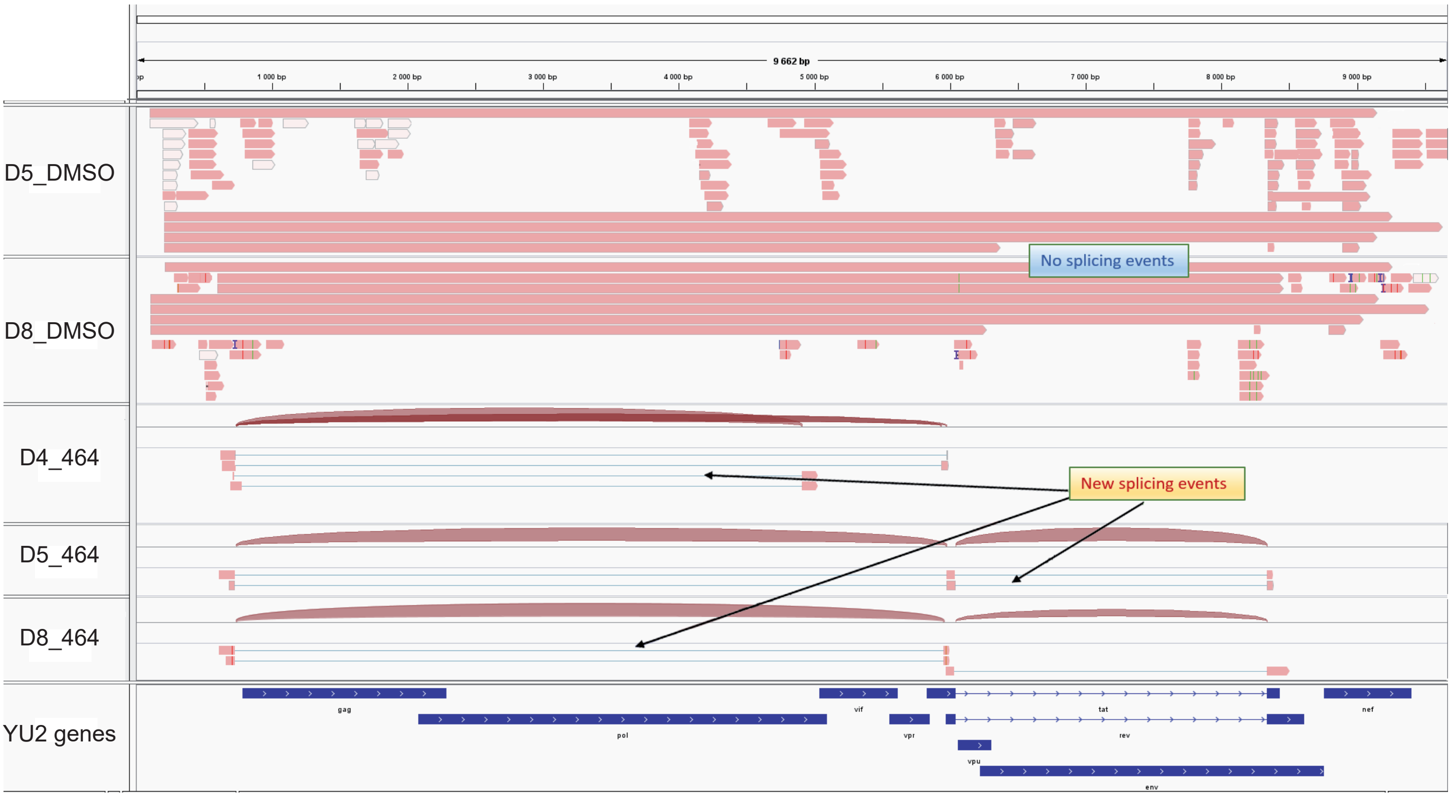

Supplementary Figure 2 : Representation of assembled contigs from two untreated samples (D5_DMSO and D8_DMSO) and three ABX464 treated samples (D4_464, D5_464 and D8_464).

Figure S3

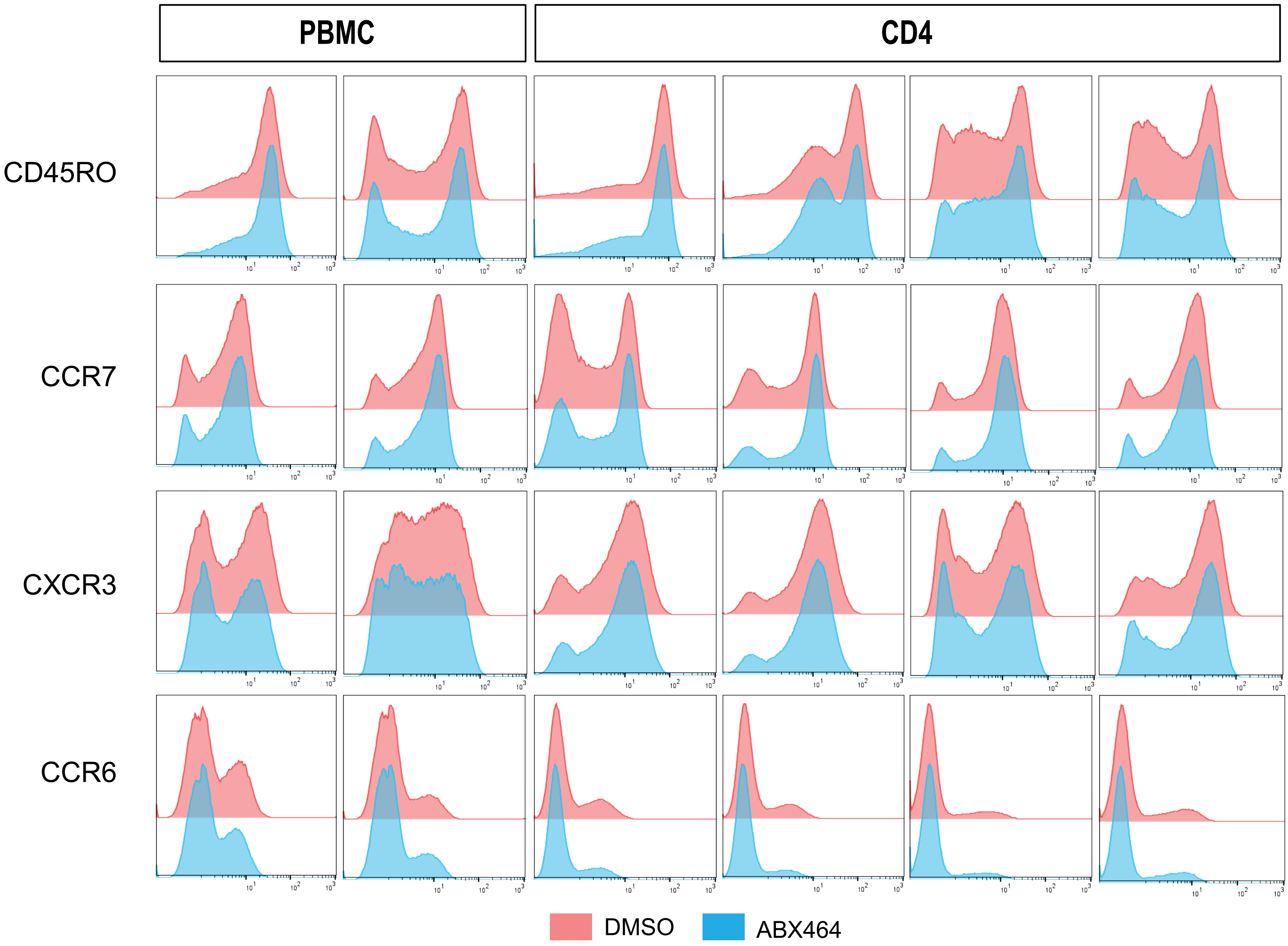

Supplementary Figure 3 : FACS analysis using CD45, CCR7, CCR3 and CCR6 surface markers of PBMCs (2 donors) and CD4 (4 donors) both untreated (red) and treated with ABX464 (blue).

Figure S4

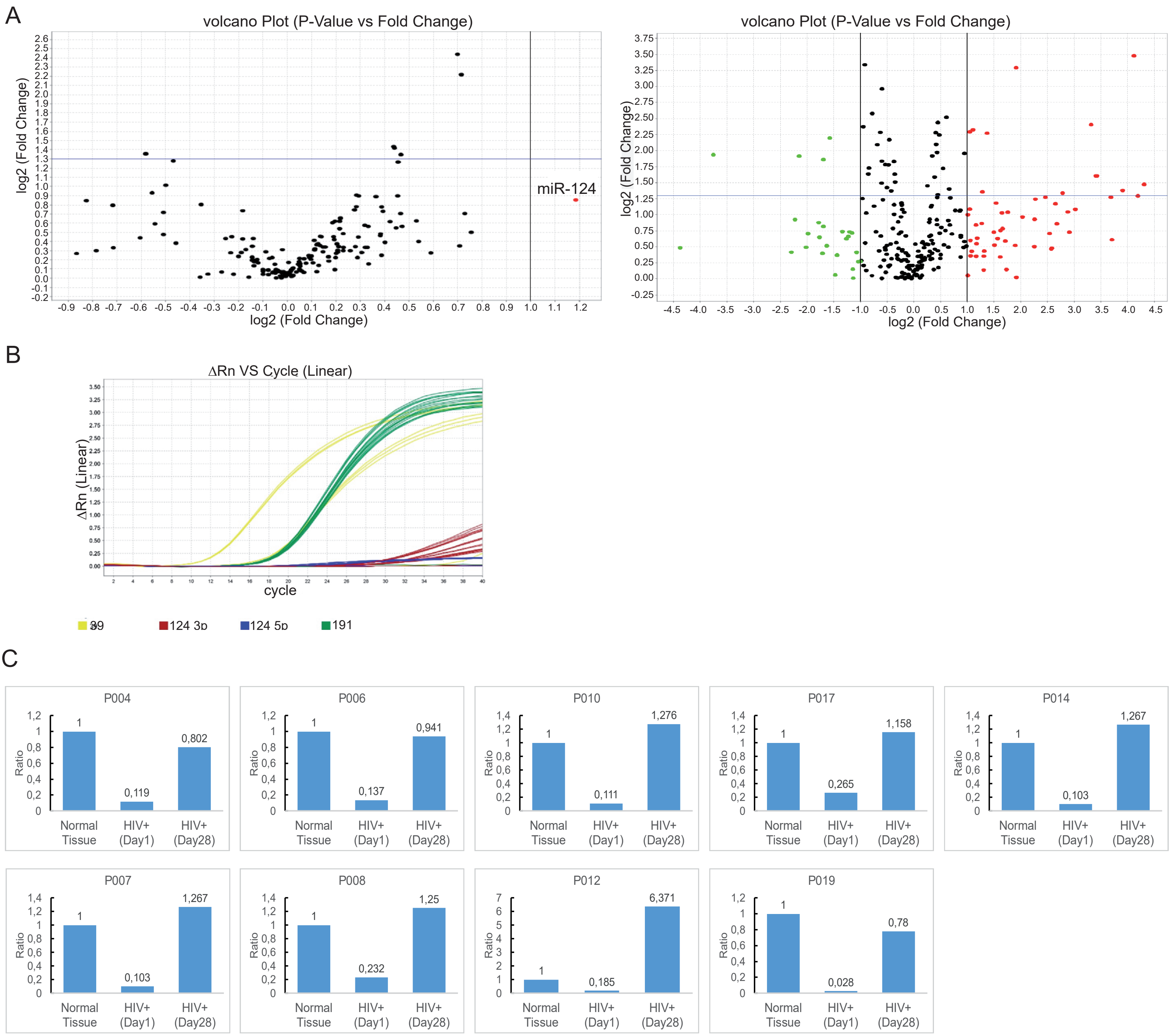

Supplementary Figure 4 : Analysis of the effect of ABX464 treatment on miRNA expression.
a. miRNA expression profiling of PBMCs from 6 donors using TaqMan Low Density Array (TLDA). The volcano plots show the differential miRNA expression in PBMCs treated vs. untreated with ABX464 (left panel), and infected vs. uninfected (right panel).
b. Comparison of miR-124 expression in macrophages of 6 donors using TaqMan PCR.
c. Quantification of miR-124 in biopsies of healthy volontaries (Normal tissue), HIV-infected patients undergoing ART after 28 days of ABX464 treatment (HIV+ Day1) and 28 days later (HIV+ Day 28).

Figure S4

D

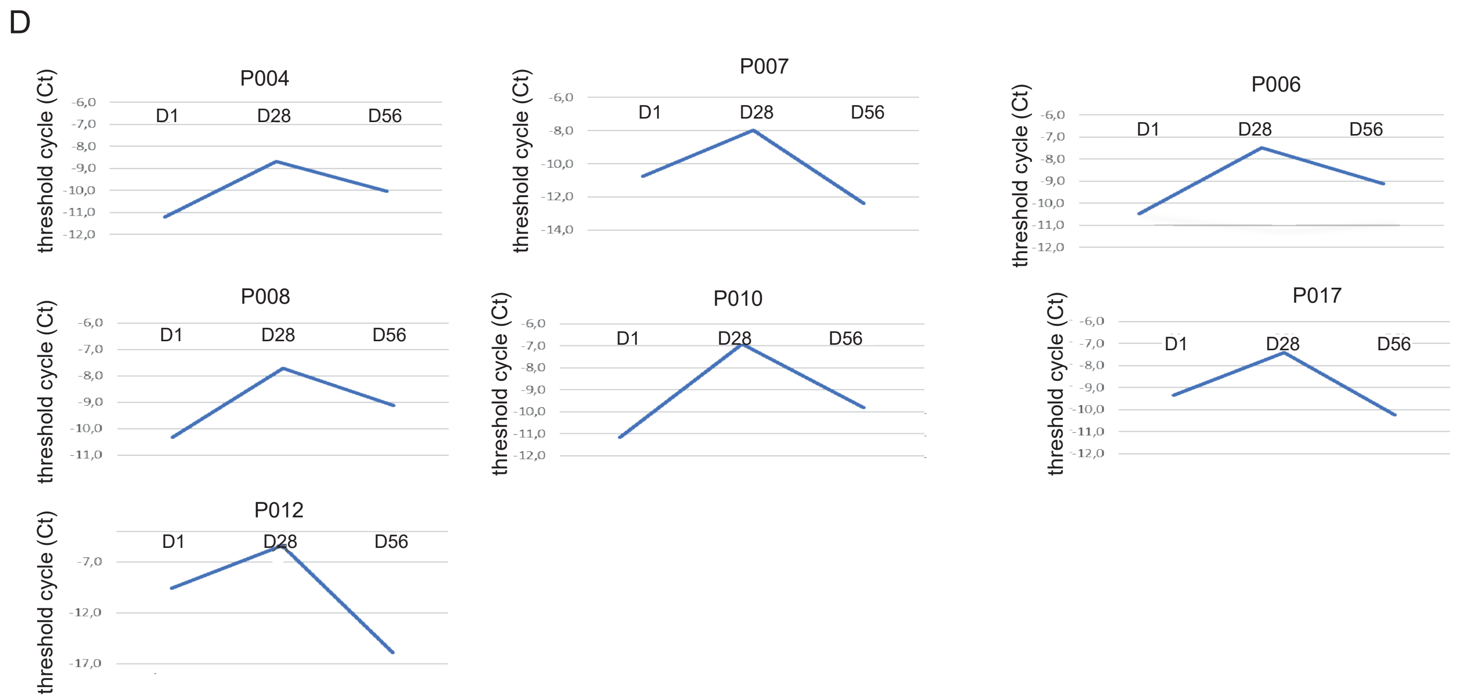

Supplementary Figure 4 : Analysis of the effect of ABX464 treatment on miRNA expression.
d. Quantification of miR-124 in biopsies of HIV patients undergoing ART at day 1 (D1) and day 28 (D28) of treatment and 28 days after stopping the treatment (D56).

Table S3

LIB names	Total reads	mir124.1	mir124.2	mir124.3
D1_DMSO	4 879 559	116164	2580	21900
D4_DMSO	30 505 238	51798	1204	5306
D5_DMSO	1 939 172	16352	1440	11709
D6_DMSO	4 385 103	13294	5358	8498
D7_DMSO	7 279 017	45480	17676	55708
D8_DMSO	8 381 026	30950	8830	18253
D1_464	7 884 109	4014	704	6439
D4_464	30 363 868	7114	4	701
D5_464	26 874 233	4748	4222	21941
D6_464	2 927 628	324	362	2542
D7_464	14 803 409	2486	2326	14460
D8_464	17 386 693	1088	5452	7772

Supplementary Table 3 : Read counts for the three genes encoding miR-124 ; *mir124.1*, *mir124.2* and *mir124.3* in PBMCs either untreated (D1_DMSO, D4_DMSO, D5_DMSO, D6_DMSO, D7_DMSO and D8_DMSO) or treated with ABX464 (D1_464, D4_464, D5_464, D6_464, D7_464 and D8_464).

Table S4

	5’ ss sequence	MAXENT score	3’ ss sequence	MAXENT score
Consensus	CAG/guaagu	10.86	ugucccuuuuuuuuccacag/CUG	12.6
Exon1-Exon2 ss	GAG/guaaag	9.65	uccuccuuuccuuuccucag/GAG	11.46
Exon2-Exon3 ss	GCG/gcggga	-4.66	uccuccuuuccuuccucag/GAG	11.46

Supplementary Table 4 : Prediction of splice site strength using maximum entropy (MaxEnt).
MAXENT scores of 5’ splice sites and 3’ splice sites at the exon1-intron1-exon2 (Exon1-Exon2 ss) and exon2-intron2-exon3 (Exon2-Exon3 ss) boundaries.
